# Environmentally sustainable surgical systems

**DOI:** 10.1136/bmjgh-2024-015066

**Published:** 2024-11-07

**Authors:** Virginia Ledda, Adewale Adisa, Fareeda Agyei, Lucy Caton, Christina George, Abdul Ghaffar, Dhruva Ghosh, Nadine Hachach-Haram, Parvez David Haque, J C Allen Ingabire, Laura Kudrna, Elizabeth Li, Craig McClain, Dmitri Nepogodiev, Faustin Ntirenganya, Mark G Shrime, Iestyn Williams, Aneel Bhangu

**Affiliations:** 1 NIHR Global Health Research Unit on Global Surgery, Institute of Applied Health Research, University of Birmingham, Birmingham, UK; 2 Department of Surgery, Obafemi Awolowo University, Ile-Ife, Nigeria; 3 Department of Paediatric Surgery, Komfo Anokye Teaching Hospital, Kumasi, Ashanti, Ghana; 4 Department of Anaesthesia, Christian Medical College and Hospital, Ludhiana, Punjab, India; 5 Department of Community Health Sciences, The Aga Khan University, Karachi, Pakistan; 6 Department of Paediatric Surgery, Christian Medical College, Ludhiana, India; 7 Department of Plastic Surgery, King’s Health Partners, Guy's and St Thomas' NHS Foundation Trust, London, UK; 8 Department of General Surgery, Christian Medical College and Hospital, Ludhiana, Punjab, India; 9 Department of Surgery, University of Rwanda, University Teaching Hospital of Kigali, Kigali, Rwanda; 10 Department of Anesthesiology, Perioperative and Pain Medicine, Boston Children’s Hospital, Boston, Massachusetts, USA; 11 Program in Global Surgery and Social Change, Harvard Medical School, Boston, Massachusetts, USA

**Keywords:** Surgery, Global Health

## Abstract

Surgeons, anaesthetists, wider surgical teams and hospital managers are a large global group that has the capacity and power to play a leadership role to contribute to change. Hospitals are a good target for improvement since they are centres of communities, linking together surrounding healthcare facilities and influencing wider determinants of the environment. District and rural hospitals are good sites to start since they serve large populations, have the least sustained energy and clean water supplies and will benefit most from quality improvement. Within hospitals, surgeons and surgical pathways are the ideal places to start decarbonising healthcare. Surgery is a high-resource activity, but it focuses on one patient at a time, allowing measures to be introduced, and their effects closely monitored. Through a mass movement, surgical teams should be able to influence policy-makers for healthcare and industry supply chains, amplifying their effect. This article describes how we can make personal, professional and organisational changes to start creating impact. Change can be hard, especially in healthcare, so this new community needs to blend carbon literacy and behavioural change techniques for success. The article is focused on the front-line team and written by clinician experts in behavioural change and sustainable practice. As such, it will not tackle the technicalities of sustainability and carbon accounting. It intends to challenge individual readers to start making changes now, and to challenge systems leaders to start making larger-scale changes urgently.

**Summary box**

- Within the hospital, various pressure groups, including surgeons, anaesthetists and theatre and hospital managers, can work together and leverage their role to guide implementation of environmentally sustainable practices.
- Sustainability in surgery can be defined to include four key areas of reliable capacity, environmental care, social responsibility and financial accountability.
- Front-line clinicians and healthcare managers need knowledge of behavioural change techniques, to create lasting change when putting into place climate protective interventions.
- Some changes need senior management and policy-maker buy-in, for which a triad of clinical, carbon and cost evidence is needed.
- Introducing renewable energy to district and rural healthcare sites in the Global South may have the double benefit of increasing capacity and reducing reliance on diesel generators while saving overall costs.

## Introduction

 Climate change has been recognised as a threat to human and planetary health. The latest trends in planetary ‘vital signs’, those factors monitored to assess the status of the planet, including greenhouse gas emissions and energy consumption, have prompted scientists worldwide to declare a climate emergency.^1^ The WHO described climate change as a significant threat to human health, with 250 000 additional deaths per year expected between 2030 and 2050.^2^ The Convention of Parties (COP), representing all governments under the United Nations Framework on Climate Change, has met annually since 1995 and established the Paris Agreement in 2015, widely regarded as the most concrete effort to address climate change.^3 4^ However, due to its lack of ambition and the insufficient pledges from countries worldwide to fulfil the agreement, this alone is unlikely to represent the answer to climate change.^5^ Organisations and individuals on the front line have the power to implement environmentally sustainable measures to mitigate climate change. Within the hospital, various pressure groups, including surgeons, anaesthetists and theatre and hospital managers, can work together and leverage their role to guide change towards sustainability in their institution.^6^ Through a mass movement, they should be able to influence policy-makers for healthcare and industry supply changes, amplifying their effect. To ensure change is sustained through time, this new community needs to blend carbon literacy and behavioural change techniques for success.^7 8^

Hospitals are a good target for improvement since they are centres of communities, linking together surrounding healthcare facilities and influencing wider determinants of the environment. They are increasingly recognised as anchor institutions, representing large organisations which are rooted within surrounding communities: changes within these institutions can provide significant benefits to the local population, other than those resulting from the provision of healthcare.^9^ In the case of environmental sustainability, for example, implementing sustainable interventions within hospitals, such as reducing the use of single-use items, can reduce the carbon impact of that hospital: less single-use items imply reduction in the volume of waste and reduction in the transport of those items to the hospital. More broadly, however, the education provided to the staff working in the hospital to sustain the intervention can lead to raised awareness of climate change and sustainability. From the staff within the hospital, this can then filter through to the wider community, with the development of ‘green communities’, more likely to engage in sustainable behaviours. In order to achieve meaningful impact, integrated approaches to change should be adopted within anchor institutions and organically implemented. Anchor institutions could also join and share strategies, in order to form networks of change within communities. The concept of anchor institution was invented in the USA and widely adopted in high-income countries including the UK.^10^ This model has gained popularity in countries of the Global South, where universities are increasingly recognised as anchor institutions for the surrounding communities.^11^,^13^ Worldwide, district and rural hospitals are good sites to implement sustainable measures to benefit communities, as change within these could have a significant beneficial impact. In fact, they serve large populations, have the least secure energy and clean water supplies, less robust finances and will benefit most from quality improvement.^14^

**Figure 1:**
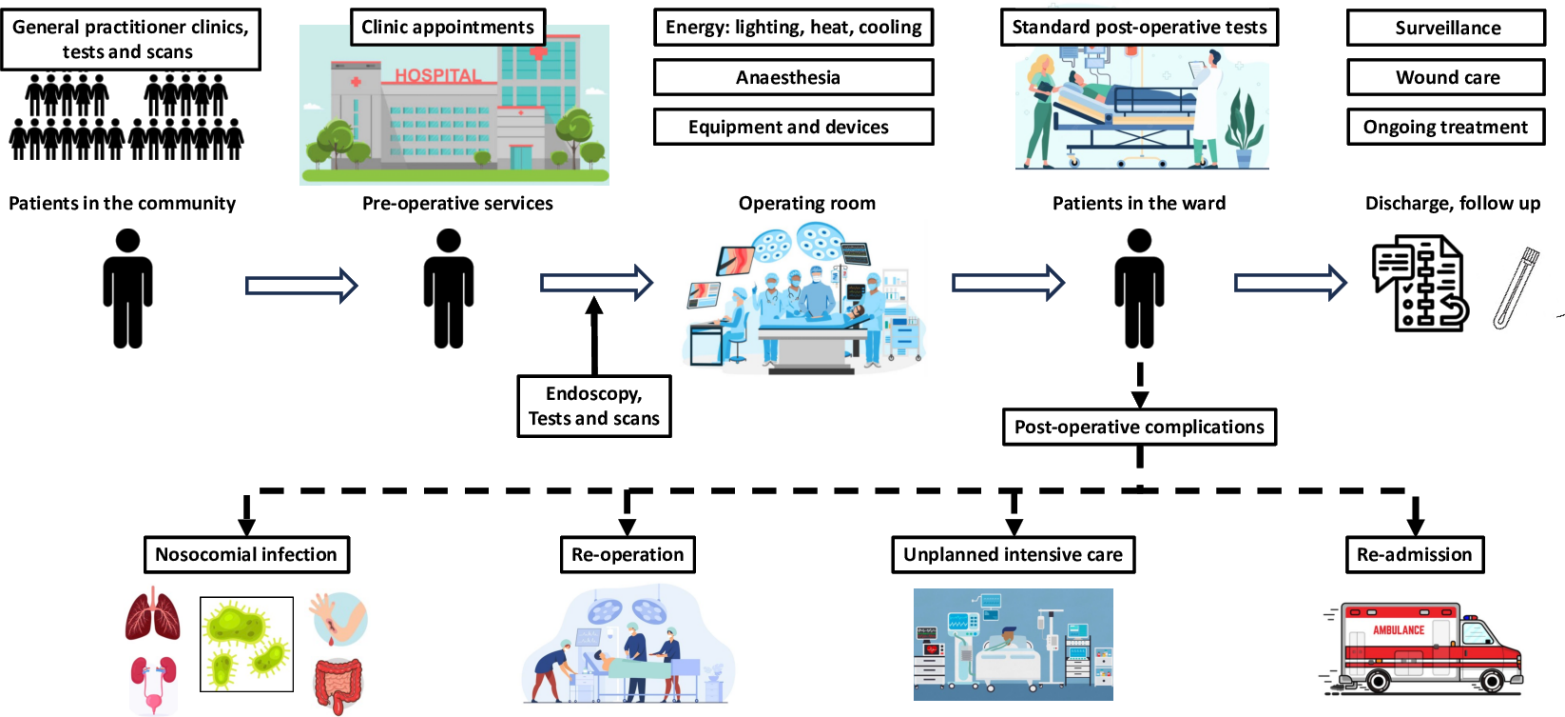
Environmental hotspots in the surgical patient’s whole pathway.

Additionally, climate change affects human health, by causing an increase in cardiovascular, respiratory and vectorborne diseases, among others.^15^ It also has a significant effect on social determinants of health, leading to an increase in population poverty and loss of habitation.^15^ As such, health systems and hospitals have a responsibility to implement sustainable interventions to mitigate the effects of climate change on the population.^16^ Within hospitals, operating theatres and surgical pathways represent a starting point for the decarbonisation of health systems. In fact, surgery is a high-resource activity with numerous environmental hotspots (figure 1), but it focuses on one patient at a time, allowing sustainable measures to be put in place, and their effects closely monitored. Those found to be most effective can be expanded beyond surgery to the wider hospital setting.^17^ Surgery is heavily reliant on stable electricity supplies, clean water and supply chains, so surgeons driving change for their patients will deliver wider benefits across the whole hospital.

This article aims to provide a comprehensive summary of challenges and opportunities in delivering environmentally sustainable surgery, addressed to the lay readers and clinicians wishing to embark on a sustainability journey. The article is focused on the front-line team and written by clinician experts in behavioural change and sustainable practice. As such, it will not tackle the technicalities of sustainability and carbon accounting.

### Government targets

The UN’s Intergovernmental Panel on Climate Change (IPCC) 2018 and 2022 reports indicate that CO₂ emissions need to be reduced by 45% by 2030 to limit global temperature rise to 1.5° by the end of the century.^18 19^ The world is currently on track to attain 10% increased emissions by 2030. Even if the combined climate pledges of 193 Parties under the Paris Agreement were carried out, this would still put the world on track for 2.5° warming by the end of the century.^20^ During the COP28 meeting, the parties achieved an agreement which softened the language to ‘phasing out’ fossil fuels, with no timeline or criteria for targets.^21^ Overall, without an extensive shift in global policy and potentially destabilising vulnerable economies of lower-income and middle-income countries, these goals are unlikely to be achieved. We need to drive front-line and managerial change.

### Defining sustainability

Sustainability is a broad term which encompasses various sectors of society. These include:

1. Reliable capacity: The ability to provide early elective care while ensuring training needs are met and enough healthcare professionals are adequately trained.
2. Environmental care: Reduction of environmental impacts by switching from single use to reusable items, optimising waste management and implementing reliable electricity and clean water in the Global South.
3. Social responsibility: Supporting ethical capacity building, slavery-free and lower carbon supply chains, and meeting wider community needs.
4. Financial accountability: Providing cost-effective care, which is financially feasible given the income and resources of an organisation.^22 23^

This article will focus on environmental sustainability and the ways that this can be implemented within surgical systems.

### The need for carbon literacy

Carbon literacy indicates the knowledge, awareness and understanding of the effects of human activities on the climate. This encompasses more theoretical knowledge on the science behind climate change, and more pragmatic skills, such as the evaluation of interventions to adopt in practice.^8^

Critically, healthcare workers represent a highly trusted profession among their patients and the community at large.^16^ By enhancing their knowledge and expertise in carbon literacy, they could improve patients’ awareness of climate change and its effects on health, benefiting the wider community.^16^

Surgeons and healthcare teams also need to improve their pragmatic skills in carbon literacy. This will enhance their ability to select which interventions to put in place, in both personal and professional settings. Without this, we are subject to industry bias and potentially false information. Although resources provided by the Carbon Literacy Project and the Centre for Sustainable Healthcare are good starting points, the surgical community worldwide would benefit from a dedicated learning resource.^24 25^

Time-consuming and expensive lifecycle assessments (LCAs) which, in surgical care, look at the complete environmental impact of a defined episode of care including production, transport and implementation of equipment and energy used to deliver care, will not be suitable for most people to conduct or interpret.^26^ Individuals will need an understanding of how to select interventions to implement in the future. For example, they should be upskilled to be able to quickly understand which interventions have niche benefits versus large benefits for carbon reduction and environmental gain. Alongside this, the academic community should develop better-standardised reporting for carbon-based outcomes, as currently reporting standards are non-comparable between studies.

### Changing behaviours

A shift towards environmentally sustainable practices, at both front-line and managerial levels, will often require successful implementation of behavioural change interventions. Behaviour change frameworks that synthesise and combine literature across individual, organisational and systems levels are promising because they take the broadest possible view of the range of factors that promote or detract from successful interventions.^27^,^29^ In designing and evaluating interventions, careful attention should be paid to the outcomes they influence; ideally, outcomes that have headroom for improvement because there are substantive gains to be made, are measurable and include both sustainability and safety. Evaluations that identify generalisable processes and mechanisms will be best placed to facilitate the diffusion of interventions across a range of contexts. We need to develop a rich understanding of what influences behaviour in these organisational contexts. This in turn can inform the design of interventions that stand a strong chance of achieving behaviour change, through, for example, education, enablement, incentives and so on.

There are many examples of effective behaviour change interventions in global health. These include targeting antibiotic prescribing with education, training and enablement through mechanisms like feedback and improving contraception usage by providing information, contraceptives and including male partners.^30 31^ It can be tempting to design a large, expensive and complex intervention to address multiple individual, organisational and system-level factors. However, in many cases, it is important to begin modestly. This draws on insights from behavioural economics that small changes can have big effects.^32^,^34^ A large and expensive intervention may not be effective or financially feasible. Instead, it is usually more effective, and cost-effective, to begin with a smaller intervention, such as one drawing on a local quality improvement initiative. This approach is used globally, including recently in a pilot in Tanzania about adolescents accessing sexual and reproductive health services.^35^ Any successful pilot intervention can be tested with learning feeding into adaptations for the next test. A new trial with the adapted intervention can then be rolled out in new contexts and settings with the test, learn, adapt cycle repeating again.

### Selecting areas to change

In order to tackle climate change within health systems, implementation of environmentally sustainable interventions needs to occur urgently, although individuals and institutions struggle with knowing where to start. By improving carbon literacy and understanding of behavioural change, many front-line practitioners will be able to identify areas to start changing.

Professional and organisational change requires a combination of individual efforts, team achievements and management buy-in. The Eco-Checklist by Practice Greenhealth and The Green Surgery Checklist, by the UK’s Royal Colleges of Surgeons, provide a starting point with recommended actions to be taken to implement sustainability in hospitals and operating theatres.^36 37^

While there is currently little evidence on the adoption of these checklists in the Global South, studies show that healthcare professionals are interested in the topic and keen to implement interventions within their practice.^38 39^ Efforts have been made to identify effective practices which could be implemented by front-line teams in the Global South.^30 40^ Industry also has a vital role, both through their supply chains and as global enablers.

Currently, these are some of the interventions used by this review’s coauthors, which the surgical teams can put into practice:

- Opening equipment only when needed during an operation, rather than at the start.
- Performing hernia surgery under local anaesthesia.
- Avoid clean waste going into infectious waste bags.
- Avoiding the use of harmful anaesthetic gases.
- Use single-use personal protective equipment only when necessary.
- Switching lights and electricity when theatre is not in use.

**Figure 2:**
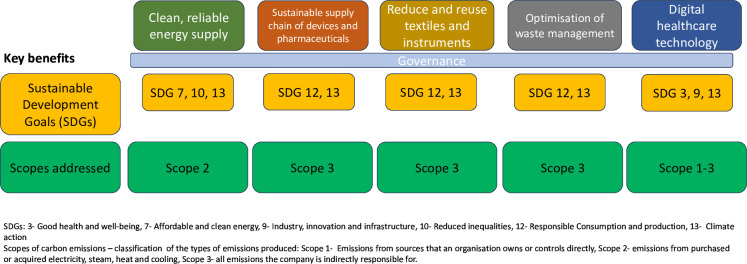
Key domains and vanguard projects for to achieve environmentally sustainable surgical systems mapped to Sustainable Development Goals (SDGs) and scopes of carbon emissions.

Below, we outline key domains and vanguard projects that can be put in place within operating theatres or by surgical teams (figure 2). Once established, these principles can be rapidly adapted and applied to the rest of the hospital and the wider patient pathway.

### Energy

Energy-saving interventions can be introduced in operating theatres to limit the emissions related to Heating, Ventilation and Air Conditioning (HVAC). These might include the installation of motion-sensing light switches, energy-efficient HVAC systems or the rationalisation of medical equipment (such as choosing an X-ray machine with a lower current output).

The provision of secure energy remains a challenge worldwide, affecting the delivery of surgical care. Renewable resources allow the provision of clean, reliable energy. The community-level benefits of solar power are clear. A randomised controlled trial of a solar lighting switchover intervention in communities in Uganda led to a significant increase in quality of life, a decrease in household lighting costs, improved educational performance and increased household safety—all in a cost-effective manner.^41^

On the supply side of the healthcare equation, sustainable energy is also an enabler of universal access to healthcare.^42^ Not only is it necessary in the fight against current disease burden, but reliable energy provision also increases an institution’s ability to attract a health workforce, to implement the information systems necessary to track and reduce adverse outcomes and to establish a robust public health system.^42^

Reliable energy access is especially important in surgery, where lapses in energy can directly lead to patient mortality.^43^ Perhaps most famously, the cuts in power at Memorial Hospital in New Orleans during Hurricane Katrina led to widely publicised deaths.^44^

Reports from Uganda also suggest that between 150 and 300 patients per year die due to power outages.^43^ The installation of solar panels at a district hospital in Sierra Leone was associated with a halving in paediatric inpatient mortality.^45^

In addition to benefits for individual patients, switching to solar-powered oxygen generation is also likely to be cost-effective in resource-constrained settings.^46^ The perception that solar energy is less reliable than other sources is unfounded. Research has shown that, like generator-powered hospitals, those powered with solar energy alone had higher failures during the day, when loads were higher, but overall had fewer failures than generator-driven comparators.^43^ Solar energy might, however, require the installation of batteries, with increased costs for initial installation and maintenance.

Projects to implement solar and other types of renewable energy (including wind power and mini-hydroelectric power) in healthcare facilities should be expanded. Evidence to assess the scale of impact in sites where electricity is currently not reliable (eg, safe birthing, more operations, more vaccines) and in sites where diesel generators are used (eg, reduction in emergency generator use and diesel purchasing) will support long-term investment.

The implementation of solar and renewable energy in hospitals could strategically represent both a mitigation and adaptation measure, especially for areas in the Global South, more vulnerable to the effects of climate change. However, further research is needed to identify the barriers and facilitators to implementing renewable energy across different resource settings. Future studies should also aim to identify key applications and prioritise areas and services that should be supplied by secure and reliable energy provision, to optimise its benefit for the hospitals and the community at large.

To ensure the potential advantage of these initiatives is fully exploited, energy-saving initiatives should also be explored, including the installation of LED lighting and the optimisation of hospital infrastructure to improve insulation and prevent dispersion. These initiatives might be difficult to implement in resource-constrained settings; however, the need and feasibility should be explored, as good-quality evidence will help secure funding opportunities.

### Reduce and reuse

The reduce and reuse principle encompasses a wide variety of interventions in the operating theatre, from reducing the number of items opened during an operation to rationalising the instrument trays or reducing the packaging of items like surgical devices

In the Global North, much equipment has become single-use, whereas in the Global South, reuse is still common.^47^ Sterilisation and reuse of supplies and devices is considered one of several frugal innovations, strategies that allow the provision of healthcare in resource-constrained settings.^48^ These innovations allow the delivery of good-quality care, with a reduction of financial and environmental costs, and as such, represent an opportunity for South-North learning.^49^

Reducing the amount of equipment on sterile trays has been proven to be a clinically safe and effective intervention, supported by the evidence from LCAs and representing a good starting point towards achieving environmentally sustainable operating theatres.^50^

Some interventions require a wider variety of stakeholders to be successfully implemented. Reducing the amount of packaging can lead to an important reduction in greenhouse gas emissions during manufacturing but also in production of waste.^30^ This requires a concerted effort from the front-line, theatre management and industry teams and likely a longer time to come into action.

**Figure 3:**
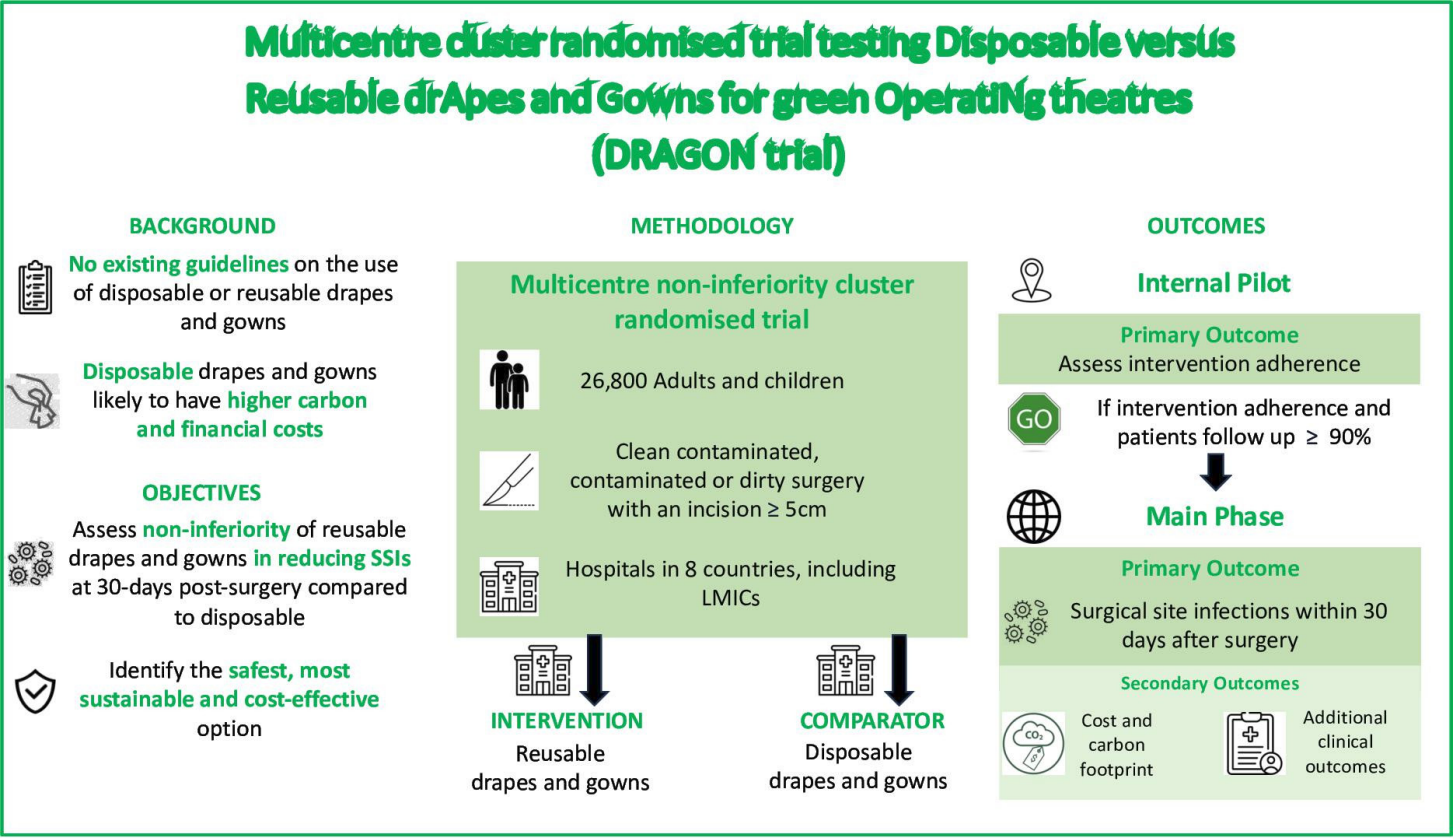
DRAGON trial infographic. LMICs: Low- and middle- income countries.

Switching from disposable to reusable textiles is also an important ‘reduce and reuse’ intervention. Both types of textiles are used in operating theatres globally and no strong evidence exists to support the use of one or the other.^51^ The DRAGON trial (Multicentre randomised trial testing Disposable vs Reusable drApes and Gowns for green OperatiNg theatres) is a major cluster randomised, non-inferiority trial testing reusable versus disposable drapes and gowns, including 26 800 patients in eight countries. The trial, due to launch in the coming weeks, will provide high-quality, pragmatic evidence with regards to clinical safety, cost-effectiveness and carbon impact (figure 3).

Industry should be leveraged to make as much equipment as possible reusable, and theatre teams should reuse, where safe and feasible. There has been an emergence of specific companies that can reprocess surgical devices, although safety concerns around infection still exist and further research is warranted.^30^

While reduce and reuse are key principles to reach sustainability, it is important to consider them within a wider picture, and ensure they are matched by good-quality evidence. For example, the CHEETAH randomised trial proved that changing gloves and instruments prior to wound closure reduces surgical site infection and is likely to reduce overall carbon expenditure.^52^

#### Reduce and reuse in anaesthesia

Reduce and reuse are also considered guiding principles to sustainable change in anaesthesia. A recent paper that came out of the World Federation of Societies of Anesthesiologists ad hoc sustainability group highlights a number of these opportunities.^53^ These recommendations came about through a Delphi process and highlighted seven areas that promote and maintain environmentally conscious anaesthesia care. The recommendations involve consciously adapting sustainable practices including drugs with better environmental profiles, minimising waste, and education, research, and QI initiatives around sustainable practices.^53^ Practically speaking, this may involve having clear strategic initiatives at the departmental and hospital levels focused on sustainable perioperative practices.

Removing desflurane from clinical practice is a step several hospitals are undertaking.^54^ Nitrous oxide, with its high global warming and ozone depleting potentials (and long atmospheric lifetime), is a next good target since up to 98% is wasted from leaks in supply systems.^55^ Nitrous oxide manifolds are increasingly being phased out in favour of canisters, and new anaesthetic machines now use medical air instead of nitrous oxide.

These initiatives must be undertaken through the lens of patient-centred care.^30^ There is much literature discussing minimal environmental impact without mention of how that affects patient outcomes. As we move into the next phase of adapting to climate change, we are compelled to combine the concerns of environmentally sustainable perioperative care with ensuring patients receive high-quality care.^56^

### Waste management

Operating theatres are resource-intensive environments, producing proportionally more waste than other areas of the hospital. Waste disposal practices vary greatly across the globe. According to the level of contamination, healthcare waste is segregated into different streams and processed appropriately to follow waste management guidelines. Usually, a higher degree of contamination requires additional management with alternative treatment (autoclaving, shredding) or higher temperatures, and an overall higher carbon footprint.^57^ For this reason, correct segregation and processing of waste is paramount to improve the sustainability of operating theatres.^7 57^ In the Global South, current waste managing practices are varied, and in some countries, these are not regulated by national guidelines.^58^ Incineration is largely favoured as the method of waste processing, as this leads to the rapid reduction of the volume of waste, and, in some cases, it allows production of energy.^59^ However, safe, regulated infrastructures are not always available in low-resource settings, with incineration taking place in major landfills, or even directly at the hospital site.^59 60^ This has health implications, with the uncontrolled release of methane and hydrocarbons, leading to the increase of respiratory and cardiovascular diseases in the local communities.^59^

The lack of regulated waste segregation also represents a risk for healthcare and waste workers, as well as the rest of the community, with the exposure to infections and injuries.^61^ The WHO advocates the introduction of a ‘three bin’ system, separating waste into non-infectious or general, infectious and sharp. The waste should also be regularly collected and accurately labelled.^61^

To achieve this, adequate education and training of the staff, suitable logistics and appropriate tendering contracts are required.^62^ Interventions in this field should incorporate behavioural change models to ensure barriers and facilitators are understood and appropriately managed, to ensure sustainability of the interventions through time.^8^

While recycling has been shown to have a limited effect in reducing the environmental impact of operating theatres, a recycling waste stream should be available and optimised in every hospital, as this would reduce the amount of waste going for incineration.^63^ The presence of private companies, which often differ among hospitals, represents a challenge in the field, with an important difference in the types and amounts of materials that can be recycled at different sites. Hard blister packs (in which much surgical equipment comes packaged) are a good target for wide-scale recycling. The plastic is clean and high value and can be readily recycled. It represents a single target for teams to focus on, and although the dent in carbon production will be small, it will prepare surgical teams for further change. However, it is dependent on ensuring a waste contractor is available to take and recycle such plastic.

Significant challenges exist, for example, ensuring only clean recyclable material enters the recycling chain and ensuring the quality of landfill for a household waste stream in places where civil environmental standards may not be enforced.

Circular economy of plastic has already been implemented in low-income and middle-income countries (LMICs), with discarded plastic being repurposed for retail.^64 65^ While this is a positive process, especially in resource settings, it is important that the environmental sustainability approach is well integrated with health policies. As such, it is important to gain a deep understanding of the health effects of repurposing waste plastics, and that policies and guidelines are in place to avoid negative health effects of the process.^65^

### Digital innovation

With five billion people lacking access to safe surgery and 143 million additional surgical procedures needed annually in LMICs, there is an estimated shortfall of 10 million health workers by 2030, mostly in LMICs.^66 67^ Improving health service coverage and realising the right to access the highest attainable standard of health is dependent on the availability and quality of staff.^66^ A new model needs to be established, both now and sustained in the future, that enables equal distribution, consistent delivery, dynamic scalability and robust cost efficiency.

Digital change can encompass the whole patient pathway, from referral through to surgery. In Rwanda, mobile health is now used to access primary care services.^68^ In many countries, telemedicine can reduce the need for travel to many routine appointments (in the UK, 5% of daily traffic is due to healthcare). The use of telephone consultations and smartphone assessments has been proven to be valuable in postoperative consultations, and to diagnose surgical site infections.^69 70^ Within the operating theatre, cloud-based software is evolving the analogue environment of the operating room into a digitally connected ecosystem, acting as a blueprint for the future of healthcare delivery.^71^ It is helping to provide high quality and sustainable training and mentorship for health workers and also support expansion of employment to support these programmes.^72^

In the field of surgical technology advances, robotic surgery has gained popularity, presenting new solutions to the barriers of laparoscopic procedures. The number of robotic procedures increased 8.4-fold in the USA between 2012 and 2018, but the uptake of robotic surgery worldwide is not homogeneous, largely due to its costs and lack of trained staff.^73 74^ While collaboration and financial support from HICs might bridge the existing disparity, robotic procedures were associated with higher greenhouse gas emissions and waste weight than their laparoscopic counterparts, with the main hotspot identified as the production of disposable items.^74 75^ Additionally, the clinical benefits of robotic surgery do not yet appear to outweigh the higher environmental impact.^75^ The evidence in this field comes from studies conducted in high-income countries. As robotic surgery gains popularity and is adopted in other parts of the world, further evidence is required to gain an understanding of its environmental impacts in low-resource settings.^74^

### Supply chains

One of the larger areas ripe for improving environmentally responsible healthcare lies not in direct care delivery, but in the supply chains, we depend on to get medically necessary equipment into the hands of healthcare providers. The Greenhouse Gas Protocol (https://ghgprotocol.org) has long been used to break down environmental impact of goods and processes across several sectors. It divides environmental impact into scopes.^76^ Scope 1 includes emissions produced from sources that are directly controlled by an organisation, such as a company’s fleet. Scope 2 includes emissions that originate from the production of energy that a company uses. Scope 3 includes all emissions which are not within scope 1 or 2. Supply chain emissions are largely included in scope 3.^76^ Much of the details of certain processes are sorted through the practice of LCAs.^26^ The use of the GHG Protocol has some limitations in being able to estimate the complete upstream and downstream energy requirements for certain processes. Alternative methods have been proposed to overcome these problems and limitations, but the GHG Protocol remains the most common and accepted method.^77^

Supply chains form the backbone of delivering safe and effective surgical care but are complex and involve multiple stakeholders. For surgical care to truly become more environmentally sustainable, the supply chain must be addressed, as its components are estimated to account for nearly 70% of healthcare-related emissions.^78^ It can be challenging to account for the entire supply chain comprehensively and accurately and this makes sustainable procurement by hospitals a complex process. Selecting the most sustainable product or surgical device, such as a stapler or laparoscopic instrument, as a hospital manager or as a surgeon, requires the evaluation of aspects of the manufacturing and production which are often not clearly presented to the customer. A solution might consist of a standardised carbon rating with a simple, universal and easily updated system, much like the traffic light nutritional value system on food packages. Evaluation cannot be exhaustive, as there are far too many devices to assess and life cycle assessment is extremely time and resource-consuming and can easily change. Outcomes are likely to be redundant as soon as a detailed life cycle assessment is completed. Furthermore, ethical considerations beyond the three scopes must also be considered. Switching to sustainable supply chains is costly, time-consuming and difficult to monitor. Change can be driven by customer demand and supply chain transparency. The urgency and magnitude of this challenge mean that we must move fast and remain adaptable along the way.

### Research and policy change

There are many other areas that can be addressed on the road to sustainability, and local teams and policy-makers need to be able to prioritise their own needs. Ultimately, implementation of sustainable practices and decarbonisation of healthcare requires effective policy change, which is tailored to different contexts and communication among front-line teams, researchers and policy-makers.^79 80^ In order for an intervention to reach the stage of policy and guideline change, this needs to be underpinned by high-quality evidence, which includes a triad of clinical data, carbon data and economic costs. In fact, sustainable interventions should still ensure safe, good-quality surgical care is provided to patients.^30 81^ Carbon and economic data may be best modelled and presented at national level to drive change. Ultimately, such data can be used to leverage national policy changes and contribute to global discussions and decisions for safe, sustainable and strong health systems.

### Future areas of research

This paper aims to illustrate the areas for improvement to achieve the delivery of environmentally sustainable surgery. Learning in this area is growing rapidly, but numerous gaps in knowledge still exist, which should be bridged by adding these topics to the current body of research:

- Understanding the availability of clean and secure energy in healthcare systems across the globe, and what the barriers are to installing solar energy facilities in hospitals.
- Characterising the current global anaesthesia practices and how to make anaesthesia provision more environmentally sustainable.
- Identify the challenges in waste management practices in the Global South and how can solutions be tailored to different contexts.

Surgical teams can be at the forefront of sustainable change in healthcare, to ensure effective decarbonisation of surgical care. This is a complex process which requires healthcare workers to become empowered, with adequate knowledge and opportunities to implement change. The areas discussed within this review represent avenues for change and a starting point for front-line workers to consider when looking at improving their practice. The solution to decarbonising surgery is not universal, as each context presents a different set of challenges. Collaboration among front-line teams, hospitals and between Global North and South can help identify and tailor the most effective interventions for each setting, in the path to reach net zero.

## Data Availability

Data sharing not applicable as no datasets generated and/or analysed for this study.
